# Effects of highly leukotoxic *Aggregatibacter actinomycetemcomitans* associated with ligature-induced periodontitis on oral and gut tissues in male rats

**DOI:** 10.1590/1678-7765-2025-0731

**Published:** 2026-03-09

**Authors:** Gabriela Furian Trama Ferraz, Camila Philipe de Araujo Camilo, Ana Luisa Palhares de Miranda, Mariana Alves Soares, Carmelo Sansone, Ricardo Tadeu Lopes, Aline Correa Abrahão, Ana Paula Vieira Colombo, Carina Maciel Silva-Boghossian

**Affiliations:** 1 Universidade Federal do Rio de Janeiro Faculdade de Odontologia Departamento de Clínica Odontológica Rio de Janeiro RJ Brasil Universidade Federal do Rio de Janeiro, Faculdade de Odontologia, Departamento de Clínica Odontológica, Rio de Janeiro, RJ, Brasil.; 2 Universidade Federal do Rio de Janeiro Faculdade de Farmácia Departamento de Biotecnologia Farmacêutica Rio de Janeiro RJ Brasil Universidade Federal do Rio de Janeiro, Faculdade de Farmácia, Departamento de Biotecnologia Farmacêutica, Laboratório de Estudos em Farmacologia Experimental (LEFEx), Rio de Janeiro, RJ, Brasil.; 3 Universidade Federal do Rio de Janeiro Instituto Alberto Luiz de Coimbra de Pós-graduação e Pesquisa de Engenharia Programa de Engenharia Nuclear Rio de Janeiro RJ Brasil Universidade Federal do Rio de Janeiro, Instituto Alberto Luiz de Coimbra de Pós-graduação e Pesquisa de Engenharia, Programa de Engenharia Nuclear, Laboratório de Instrumentação Nuclear, Rio de Janeiro, RJ, Brasil.; 4 Universidade Federal do Rio de Janeiro Faculdade de Odontologia Departamento de Patologia Oral Rio de Janeiro RJ Brasil Universidade Federal do Rio de Janeiro, Faculdade de Odontologia, Departamento de Patologia Oral, Rio de Janeiro, RJ, Brasil.; 5 Universidade Federal do Rio de Janeiro Instituto de Microbiologia Rio de Janeiro RJ Brasil Universidade Federal do Rio de Janeiro, Instituto de Microbiologia, Rio de Janeiro, RJ, Brasil.

**Keywords:** Aggregatibacter actinomycetemcomitans, Periodontal diseases, Periodontal pathogen, Small intestine

## Abstract

**Objective:**

To evaluate the effects of gastric administration of the highly leukotoxic *Aggregatibacter actinomycetemcomitans* JP2 strain (Aa JP2), with or without ligature-induced periodontitis, on periodontal and small intestinal tissues in a rat model.

**Methodology:**

Forty-eight-week-old male Wistar rats were divided into four groups (n=10/group): Control (CG), Periodontitis (PG), Periodontitis and *Aa* JP2 (PAaG), and *Aa* JP2 only (AaG). Ligatures were placed in PG and PAaG on day one and maintained for 42 days. After six weeks of twice-weekly gastric *Aa* JP2 inoculation, mandibles and small intestines were analyzed using stereomicroscopy, micro-computed tomography (CT), and histopathology to assess alveolar bone loss, trabecular architecture, inflammatory infiltrate, vascular congestion, goblet cell density, and villus morphology. Statistical analyses included the Kruskal–Wallis, Mann–Whitney, and Chi-square tests for group comparisons (p<0.05), as well as Spearman’s correlation test.

**Results:**

PG and PAaG exhibited significantly greater bone loss compared to CG and AaG (*p*<0.05). Micro-CT analysis revealed reduced trabecular thickness in AaG (342.4±23.1µm) compared to PG (378.2±28.1µm) and PAaG (385.5±45.1µm) (*p*<0.05). PAaG and AaG presented higher inflammatory infiltrate scores (>751 inflammatory cells) compared to CG and/or PG (*p*<0.05). Elevated vascular congestion and/or goblet cell hyperplasia in the jejunum and ileum were observed in PAaG and AaG compared to CG and PG (*p*<0.05). Villus height (duodenum/ileum) and villus width (jejunum/ileum) differed significantly among groups (p<0.0001). Significant correlations were identified: the alveolar bone volume/total volume ratio was positively associated with duodenal vascular congestion (*rho*=0.738; p=0.037); the periodontal inflammatory infiltrate was positively associated with jejunal goblet cell counts (*rho*=0.704; p=0.007); alveolar bone loss was positively associated with both duodenal and jejunal villus height (rho≥0.762; p<0.05); and bone volume was negatively associated with both jejunal vascular congestion (*rho*=-0.696; *p*=0.003) and jejunal goblet cell counts (*rho*=-0.617; *p*=0.011).

**Conclusions:**

Gastric inoculation with Aa JP2 exacerbates ligature-induced periodontitis and independently induces intestinal morphological changes.

## Graphical abstract

**Figure:**
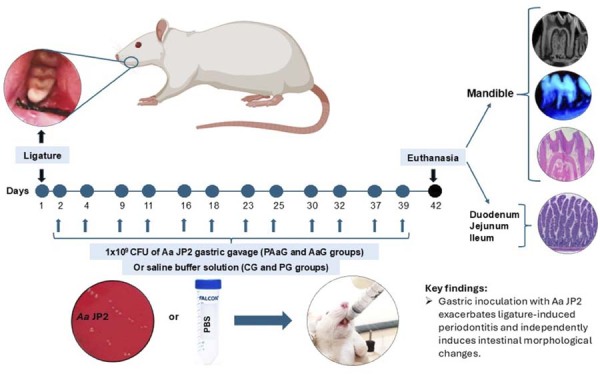

## Introduction

Periodontitis is a chronic inflammatory disease associated with a dysbiotic biofilm, characterized by a complex interplay of factors that leads to periodontal tissue breakdown and potential tooth loss.^1^ As the disease progresses, structural and metabolic shifts in the periodontal environment promote the establishment of a dysbiotic microbial community dominated by more pathogenic and virulent species.^1,2^ Among these, the highly leukotoxic JP2 clone of *Aggregatibacter actinomycetemcomitans* (*Aa* JP2) has been identified as a key pathogen in rapidly progressing periodontitis.^3,4^

The *Aa* JP2 clone exhibits numerous virulence factors, including adhesins, fibrins, lipopolysaccharide, cytolethal distension toxin, host cell invasiveness, and high leukotoxin production.^3-6^ Although non-motile, *Aa* JP2 can disseminate hematogenously or via deglutition, enabling colonization of distant tissues and organ systems.^5,7^ These pathogenic traits may explain its reported associations with systemic conditions beyond periodontitis, such as rheumatoid arthritis, hepatic disorders, and gastrointestinal diseases.^8-10^ However, although circulating *Aa* JP2 has been associated with several systemic conditions, its autotransporter adhesins have been shown to exhibit specific tropism for oral tissues.^11,12^ Furthermore, *Aa* JP2 can specifically adhere to other pathogenic periodontal bacteria.^13^

The persistent low-grade inflammatory state observed in periodontitis can cause systemic injury. This process is generalized and may contribute to tissue degeneration or aggravate pre-existing lesions.^14^ Thus, chronic systemic inflammation driven by periodontal bacteria and associated inflammatory mediators can increase intestinal permeability and the recruitment of pathogenic T cells to the intestine, inducing intestinal imbalance and inflammation.^9^ Another possible mechanism underlying the relationship between oral bacteria and intestinal disease is the ability of oral microorganisms to colonize the lower digestive or gastrointestinal system, particularly in individuals with preexisting intestinal disorders.^7,15,16^ Upon reaching the intestine, these oral bacteria may enhance the virulence of enteric pathogens, disrupt the diversity and relative abundance of the gut microbiota, and contribute to inflammation.^7,15,16^ In this scenario, even without established inflammation, an imbalanced intestinal microbiota can increase intestinal wall permeability.^17^

Animal studies involving oral–gut inoculation with *Porphyromonas gingivalis* and *Fusobacterium* spp., for instance, suggest their role in altering the intestinal microenvironment.^18^ These findings can potentially redefine periodontal therapy as a necessary systemic intervention to prevent downstream metabolic and intestinal disorders. This perspective supports a more integrated and holistic model of care, in which physicians and dentists collaborate. However, it remains unclear whether oral bacteria directly or indirectly trigger intestinal alterations, potentially contributing to metabolic disorders.^10,15,18,19^ In the case of highly leukotoxic *Aa*, a study by da Costa, et al.^19^ (2024) demonstrated that the introduction of *Aa* JP2 into the rat gut induced local immunosuppression and microbial imbalance, marked by increased pathobionts and reduced commensals, without disrupting the mucosal barrier or inducing colonic inflammation. Nevertheless, that study did not assess the compounded effects of pre-existing periodontitis. Here, we used a rat model to investigate the effects of gastric *Aa* JP2 administration, combined with ligature-induced periodontitis, on periodontal and small intestine tissues.

## Methodology

### Animal groups

This study was conducted in compliance with the ethical principles for animal experimentation established by the National Council for Control of Animal Experimentation (CONCEA) and was approved by the Ethics Committee on the Use of Animals at the Federal University of Rio de Janeiro (Protocol #065/21). The study also complies with the Animal Research: Reporting of In Vivo Experiments (ARRIVE) guidelines 2.0.^20^

Animals were monitored throughout the study for measurable clinical signs, including progressive and fast weight loss, anorexia, disabling diarrhea, dehydration, edema, abdominal enlargement, progressive dermatitis, altered hair appearance, altered posture, and self-inflicted trauma, among others. All animals were weighed at baseline, weekly, and upon euthanasia.

The sample size was calculated based on the standard deviation for duodenal villus height previously reported.^21^ A standard deviation of 72.21µm was used for the control group (A) and 44.91 µm for the test group (B), with an estimated intergroup difference of 90µm. This calculation was performed using WinPepi software (http://www.brixtonhealth.com/pepi4windows.html), which determined that 10 animals per group would be sufficient to detect this difference with a 95% confidence interval (±55.105), 90% power, and a 5% significance level (alpha).

Forty-eight-week-old male Wistar rats (220–300 g) were housed under controlled conditions (12-hour light–dark cycle) with free access to water and standard chow at the Animal Facility of the Faculty of Pharmacy, Federal University of Rio de Janeiro*.* The animals were randomly allocated into four experimental groups (n = 10 per group): CG, control group receiving gastric gavage with phosphate-buffered saline (PBS); PG, ligature-induced periodontitis and gastric gavage of PBS; PAaG, ligature-induced periodontitis and gastric gavage of AaJP2; and AaG, gastric gavage of AaJP2.

### Ligature-induced periodontitis

**Figure 1:**
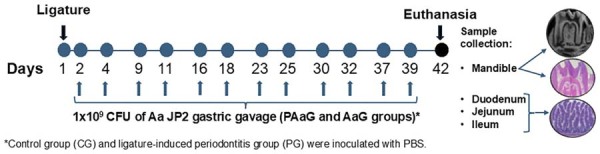
Schematic representation of the experimental timeline. Forty-eight-week-old male Wistar rats were allocated into four groups (n=10): Control (CG), Periodontitis (PG), Periodontitis + Aa JP2 (PAaG), and Aa JP2 only (AaG). Ligature placement was performed on day one in PG and PAaG. Gastric inoculations—either Aa JP2 (PAaG and AaG) or PBS (CG and PG)—were administered twice weekly from day two to 39. All animals were euthanized for sample collection on day 42.

Periodontitis was induced using a previously described method.^22^ After anesthesia via intraperitoneal injection of ketamine (80 mg/kg; Cetamin, Syntec, Tamboré, SP, Brazil) and xylazine (10 mg/kg; Xilazin^®^, Syntec), a cotton ligature was placed around the cervical region of the lower first molars bilaterally. Ligatures were installed at the beginning of the experiment in PG and PAaG and were checked periodically. They were placed on day one and maintained throughout the 42-day experimental period (Figure 1). No animals were lost due to ligature loss; therefore, no replacements were required according to the protocol.

### Bacterial culture and gastric inoculation

*Aa* JP2 (strain HK921) was cultured in 5 mL of Trypticase Soy Broth supplemented with yeast extract (BD Difco™ Bacto™ Brain Heart Infusion; Thermo Scientific, Waltham, MA, USA) for 72h at 37 °C under anaerobic conditions (80% N2, 10% H2, 10% CO2). An aliquot of the bacterial culture was then plated on Fastidious Anaerobe Agar (Thermo Scientific) containing 5% sheep blood and incubated under the same conditions.^19^

Colonies were suspended and dispersed in PBS, and the concentration was adjusted to an at 600 nm optical density (OD600 = 1), equivalent to 1×10^9^ colony-forming units (CFU)/mL. Each animal received 200 µL of bacterial suspension or PBS via gastric gavage twice weekly for six weeks. Gastric gavage was performed using a specialized 16-gauge oral feeding needle (3 cm in length, curved, with a rounded ball tip) to ensure safe passage into the esophagus and stomach without injuring sensitive tissues. Frequency of administration and bacterial concentration were based on a previous study.^19^

### Stereomicroscopic and micro-computed tomography analysis

At the end of the experiment (42 days), animals were anesthetized by intraperitoneal injection of ketamine and xylazine (240 and 30 mg/kg, respectively). The abdomen and thorax were surgically opened and small intestine segments and mandibles were harvested and either fixed in buffered formalin or processed for micro-computed tomography (µ-CT).

Small intestine segments—including the duodenum, jejunum, and ileum—were collected according to anatomical landmarks. The duodenum was separated from the jejunum at the ligament of Treitz, while the ileum was separated from the jejunum at the region characterized by an increase in diameter and mesenchyme tissue.^23,24^

Left hemimandibles were immersed in 30% hydrogen peroxide for two hours, then stained with 1% methylene blue for one minute to highlight the cementoenamel junction (CEJ), as previously described.^25^ Digital images of the buccal aspects of the first molars were captured using a stereomicroscope (Leica L8AC0) coupled to a digital camera (Leica DFC290 HD/ CLS 150) and analyzed using ImageJ software (NIH, Bethesda, MD, USA). Bone loss was quantified as the mean of three linear measurements from the CEJ to the alveolar bone crest for each root.

For µ-CT analysis, samples were scanned using a Skyscan Bruker 1173 system with the following parameters: (a) pixel size of 16 μm; (b) detector array of 2024 x 2024 pixels; (c) rotation of 0.5°; (d) X-ray voltage of 70kV; and (e) electrical current of 114 μA. Acquired images were reconstructed using Nrecon v. 1.6.10.4 and processed with DataViewer v. 1.5.0. The region of interest (ROI), encompassing the furcation area of the lower first molars, was defined across 100 transaxial slices. Following binarization, morphometric analysis was performed using ImageJ to calculate: (a) total bone porosity ratio; (b) bone volume density (%BV/TV); and (c) trabecular thickness, using the BoneJ plugin.

### Histological analysis

Right hemimandibles and small intestine segments were processed for histopathological analysis. Intestinal samples were divided into the duodenum, jejunum, and ileum, and hemimandibles were decalcified in an 18% EDTA solution prior to processing. Tissues were fixed in 10% neutral buffered formalin for 48 h, dehydrated through a graded ethanol series (absolute, 95% to 70%), embedded in paraffin, and sectioned at a thickness of 4 μm.

Hematoxylin-eosin-stained slides were evaluated by light microscopy (100x magnification). Periodontal analysis focused on the distal root of the first molar and the interproximal area between the first and second molars in buccal-lingual sections. Inflammation was graded as: score 0, mild infiltrate (fewer or no inflammatory cells, 0–450 inflammatory cells); score 1, moderate infiltrate (451–750 inflammatory cells); and score 2, severe infiltrate (>751 inflammatory cells).^21^

For intestinal morphology, the same analyses were performed for all three small intestine segments, including villus measurements, goblet cell counts, and evaluation of vascular congestion. Histological images displaying perpendicularly orientated villi were measured using ImageJ. Villus height was measured from the tip to crypt junction (mean of 10 villi/slide), and villus width was measured at the luminal extremity. Goblet cell density and vascular congestion were recorded for at least three villi per slide.^21^

### Data analysis

All samples were evaluated in each analysis, including imaging and histological analyses, by a calibrated examiner blinded to the experimental groups. Data normality was assessed using the Kolmogorov–Smirnov test. Significant differences among groups were analyzed using the Kruskal–Wallis test, and the Mann–Whitney U test was employed for pairwise comparisons. Categorical variables were evaluated using the Chi-square test. Intragroup differences in body weight gain were assessed using the Wilcoxon signed-rank test. Correlations analyses including all groups were conducted using Spearman’s rank correlation coefficient. All statistical analyses were performed using SPSS version 22.0 (IBM Corporation, Armonk, NY, USA), with the significance level set at *p*<0.05.

## Results

### Body and intestinal mass measurements

Body mass and small intestine segment measurements are presented in Supplementary Table S1. No significant intergroup differences were observed in baseline or final body mass (*p*>0.05; Kruskal–Wallis test). All groups showed significant weight gain at the end of the study compared to baseline (*p*=0.005; Wilcoxon test). No significant intergroup differences were detected in small intestine segment masses.

### Bone morphometric analysis

**Figure 2:**
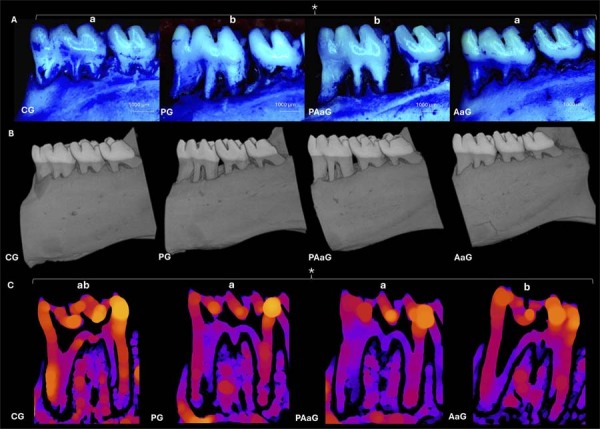
Representative stereomicroscopic and micro-computed tomographic analyses of the left hemimandible. (A) Stereomicroscopic images, (B) 3D reconstruction of micro-computed tomography images, and (C) trabecular thickness measurement. * *p*<0.05, Kruskal–Wallis test; Different letters in the same row indicate significant differences between groups, as determined by the Mann–Whitney U test (*p*<0.05). Pixel size (B, C): 7.023770 µm. CG (n=10): control group; PG (n=10): periodontitis group; PAaG (n=10): periodontitis and *Aa* JP2 group; AaG (n=10): *Aa* JP2 group.

Stereomicroscopic analysis (Figure 2A) demonstrated significant intergroup differences in bone resorption at the mesial (*p*=0.001), buccal (*p*=0.005), and distal (*p*=0.010) aspects, as well as in mean bone loss (*p*=0.002; Table 1). Periodontitis groups (PG and PAaG) exhibited significantly greater bone loss than non-periodontitis groups (CG and AaG; *p*<0.01, Mann–Whitney test). No intergroup differences were found in bone volume or trabecular volume. Bone density demonstrated heterogeneous values across groups, with no significant differences. Trabecular thickness varied significantly among groups (*p*=0.015), with AaG (342.4±23.1 µm) showing lower values than PG (378.2±28.1 µm) and PAaG (385.5±45.1 µm; *p*=0.010). Representative μ-CT reconstructions (Figure 2B) and BoneJ-processed trabecular thickness images (Figure 2C) are provided.

**Table 1:** Mean (± standard deviation) values of alveolar bone loss (mm), bone volume (µm3), trabecular volume (µm3), bone density (bone volume/trabecular volume; %), and trabecular thickness (µm), and distribution of periodontal inflammatory scores across experimental groups.

Measurements	CG	PG	PAaG	AaG	P value*
	**(n=10)**	**(n=10)**	**(n=10)**	**(n=10)**	
Mesial BL (mm)	1.4(±0.2)^a^	1.9(±0.3)^b^	2.1(±0.5)^b^	1.5(±0.2)^a^	**0.001**
Buccal BL (mm)	1.3(±0.2)^a^	1.5(±0.4)^b^	1.7(±0.4)^b^	1.2(±0.3)^a^	**0.005**
Distal BL (mm)	0.9(±0.3)^a^	1.1(±0.4)^b^	1.3(±0.4)^b^	0.9(±0.1)^a^	**0.010**
Mean BL (mm)	1.2(±0.2)^a^	1.5(±0.3)^b^	1.7(±0.4)^b^	1.2(±0.2)^a^	**0.002**
BV (µm^3^)	3.1(±0.7)	3.9(±1.4)	3.5(±1.9)	2.9(±0.9)	>0.05
TV (µm^3^)	3.4(±3.2)	2.1(±1.3)	3.3(±1.9)	3.7(±2.2)	>0.05
BV/TV rate (%)	34.4(±6.4)	32.8(±7.4)	36.4(±5.3)	38.1(±4.8)	>0.05
Trabecular thickness (µm)	365.1(± 28.7)^ab^	378.2(±28.1)^a^	385.5(±45.1)^a^	342.4(±23.1)^b^	**0.015**
*Inflammatory infiltrate*					
Mild	60%	0%	0%	0%	**0.006†**
Moderate	40%^a^	83.3%^ab^	20%^c^	25%^bc^	
Intense	0%	16.7%	80%	75%	

* Kruskal–Wallis test; † chi-squared test; Different letters in the same row indicate significant differences between groups, as determined by the Mann–Whitney U test (p<0.05); CG: Control group, PG: Periodontitis group; PAaG: Periodontitis and Aa JP2 group; AaG: Aa JP2 group. BL: bone loss; BV: bone volume; TV: trabecular volume.

### Histological findings

Periodontal inflammatory infiltrate scores differed significantly among groups (*p*=0.006, Chi-square test; Table 1). Groups exposed to *Aa* JP2 (PAaG and AaG) exhibited more severe inflammatory inflammation than non-exposed groups (CG, PG). Specifically, CG showed lower scores than PAaG (*p*=0.026) and AaG (*p*=0.043), while PG demonstrated lower scores than PAaG (*p*=0.036). Dental pulp findings are presented in Figure S1 (Supplementary Material). PG exhibited a significantly lower number of congested pulp vessels compared to PAaG (*p*=0.006).

Table 2 presents the measurements of intestinal villi. Villus height differed significantly among groups in the duodenum and ileum, while villus width differed significantly among groups in the jejunum and ileum (*p*<0.0001). CG presented significant lower villus height values in both the duodenum and ileum compared to other groups (*p*<0.05). PAaG had higher villus width values in the jejunum than all other groups (*p*<0.05). In the ileum, villus width was higher in CG compared to other groups (*p*<0.05).

**Table 2:** Mean (± standard deviation) villus measurements (mm) by experimental group.

Small intestine segments	Mean (mm)				P value*
	CG	PG	PAaG	AaG	
	(n=10)	(n=10)	(n=10)	(n=10)	
***Duodenum***					
- Height	0.26±0.01^a^	0.39±0.06^bc^	0.40±0.01^b^	0.33±0.07^c^	**<0.0001**
- Width	0.07±0.04	0.08±0.01	0.07±0.01	0.06±0.04	>0.05
***Jejunum***					
- Height	0.37±0.13	0.39±0.07	0.41±0.07	0.33±0.05	>0.05
- Width	0.07±0.02^a^	0.07±0.01^a^	0.09±0.0^1b^	0.07±0.01^a^	**<0.0001**
***Ileum***					
- Height	0.17±0.1^a^	0.26±0.05^b^	0.32±0.06^c^	0.27±0.01^bc^	**<0.0001**
- Width	0.13±0.05^a^	0.08±0.02^b^	0.08±0.01^b^	0.07±0.01^b^	**<0.0001**

*Kruskal–Wallis; Different letters in the same row indicate significant differences between groups, as determined by the Mann–Whitney U test (p<0.05); CG: Control group, PG: Periodontitis group; PAaG: Periodontitis and Aa JP2 group; AaG: Aa JP2 group.

**Figure 3:**
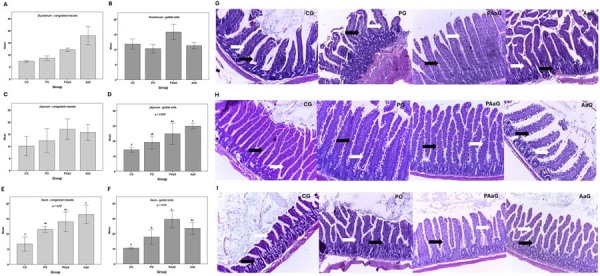
Quantification of congested vessels and goblet cells in small intestine segments. (A) Congested vessels in the duodenum; (B) Goblet cells in the duodenum; (C) Congested vessels in the jejunum; (D) Goblet cells in the jejunum; (E) Congested vessels in the ileum; (F) Goblet cells in the ileum; Representative photomicrographs of the duodenum (G), jejunum (H), and ileum (I); White arrows: congested vessels; Black arrows: goblet cells; Magnification: 100x; Hematoxylin and eosin stain. * Kruskal–Wallis test; Different letters above the bars denote significant differences between groups (p<0.05, Mann–Whitney U test). CG: control group; PG: periodontitis group; PAaG: periodontitis and *Aa* JP2 group; AaG: *Aa* JP2 group.

Duodenal vessel congestion and goblet cell counts were not significantly different among groups (Figure 3A-B). In the jejunum, no difference was found in vessel congestion among groups (Figure 3C); however, goblet cell counts differed significantly (*p*=0.012; Figure 3D). The CG group presented a significantly lower number of jejunal goblet cells than PAaG (*p*=0.043) and AaG (*p*=0.021) groups. PG also exhibited a significantly lower number of goblet cells than AaG (*p*=0.020). In the ileum (Figure 3E-F), significant differences were found for both vessel congestion (*p*=0.026) and goblet cell counts (*p*=0.017). Regarding ileal vessel congestion (Figure 3E), PG differed significantly from AaG (*p=*0.021), while CG differed significantly from both PAaG and AaG (p<0.05). For ileal goblet cell counts (Figure 3F), CG showed significantly lower values compared to all other groups (p < 0.05). Furthermore, PAaG differed significantly from PG but not from AaG (*p*<0.05). Figure 3 also displays representative histological images of the duodenum (G), jejunum (H), and ileum (I).

### Correlation analyses

Significant correlations were found between oral and intestinal parameters (Table 3; Supplementary Table S2). Strong positive correlations were observed between periodontal inflammation and jejunal goblet cell counts (*rho=*0.704; *p*=0.007), as well as between the bone volume/total volume ratio and duodenal vessel congestion (*rho=*0.738; *p*=0.037). Additionally, mean bone loss was positively correlated with duodenal villus height (*rho=*0.929; *p*=0.001), while distal bone loss correlated with jejunal villus height (*rho=*0.762; *p*=0.028). Strong significant negative correlations were also identified between bone volume and both jejunal vessel congestion (*rho=*-0.696; *p*=0.003) and goblet cell counts (*rho=*-0.617; *p*=0.011).

**Table 3:** Significant bivariate correlations between variables.

	Inflammatory infiltrate in the periodontium	BV	BV/TV	Trabecular thickness	Mean BL	Mesial BL	Buccal BL	Distal BL
***Congested vessels:***								
Duodenum	-	-	**0.738(0.037)**	-	-	-	-	-
Jejunum	-	-0.696(0.003)	-	-	0.542(0.030)	0.661 (0.005)	-	-
Ileum	0.557(0.048)	-	0.566(0.034)	-	-	-	-	-
Goblet cells in the jejunum	**0.704(0.007)**	-0.617(0.011)	-	-	-	0.527 (0.036)	-	-
***Villus height:***								
Duodenum	-	-	-	-	**0.929(0.001)**	-	-	**0.762(0.028)**
Jejunum	-	-	-	0.538(0.038)	-	-	-	-
Villus width in the ileum	-	-	0.577(0.049)	-	-	-	-	-
Final body mass	-	-	-	0.447(0.004)	-	-	-	-
Duodenum mass	-	-	-	-	0.397(0.011)	-	0.505(0.001)	0.338(0.033)
Ileum mass	-	-	-	-	0.428(0.006)	-	0.530(<0.0001)	0.401(0.010)

BL: bone loss; BV: bone volume; TV: total volume.

## Discussion

Our findings demonstrate that gastric administration of the highly virulent oral pathogen *Aa* JP2 induces significant inflammatory and vascular alterations in both periodontal and intestinal tissues, independent of ligature-induced periodontitis. This indicates that *Aa* JP2 disrupts small intestine homeostasis, even in the absence of classical periodontal bone loss. Moreover, ligature-induced periodontitis alone provoked distinct intestinal alterations, underscoring that both the presence of a specific pathogen and periodontitis itself can independently impact the gut mucosa.

In the current study design, direct inoculation of *Aa* JP2 into the stomach was intended to simulate deglutition and avoid direct contact with oral tissues. The inoculation method, as well as the frequency of administration and dosage, was based on a previous study demonstrating that this protocol induces gut microbiota dysbiosis and reduces colonic myeloid cell numbers in the *lamina propria*.^19^ Therefore, we explored the combined effects of gastric *Aa* JP2 exposure and ligature-induced periodontitis on oral and intestinal tissues, hypothesizing that this highly virulent strain would significantly impact the investigated variables. As demonstrated in another study, *Aa* adhesins exhibit specific tropism for oral tissues, enabling its translocation from gastric tissues to the oral cavity.^11^ Once established at oral sites, *Aa* JP2 virulence factors are expected to trigger a local immune response,^3,4,6,26^ characterized by increased blood supply—reflected by an increased number of congested blood vessels—and inflammatory infiltration, ultimately resulting in bone resorption. In this study, the combination of ligature-induced periodontitis and *Aa* JP2 exposure was employed to test the potential additive effect of both interventions. Only *Aa* JP2 was investigated; whether other species or strains produce similar outcomes remains to be determined.

Stereoscopic analysis revealed that vertical alveolar bone measurements in AaG did not differ from those of CG, whereas both groups differed significantly from PG and PAaG, confirming the effect of ligature-induced periodontitis. Surprisingly, only AaG presented significantly reduced bone thickness compared to PG and PAaG. Reduced bone thickness has been associated with poorer bone quality.^27,28^ The presence of *Aa* JP2 at extraoral sites may impact systemic aspects by modulating the host immune response or altering the gut microbiome balance, thereby indirectly affecting periodontal structures.^5,8,9,26^ Studies on other oral bacteria show that they may also be associated with intestinal dysbiosis, which could trigger inflammation and contribute to disease development at distant body sites.^9,19^

The observed associations between alveolar bone loss and intestinal alterations—including increased villus height, vascular congestion, and goblet cell proliferation—suggest systemic pathological interactions. These intestinal morphological changes, particularly elevated goblet cell counts and vascular congestion, are consistent with an inflammatory state. Regarding villus height, CG exhibited lower values compared to the other groups. This contrasts with previous reports showing no ileal changes in periodontitis-only models.^21^ Villus morphology is known to reflect enterocyte turnover and positioning, with inflammation typically causing villus shortening rather than elongation, as demonstrated in an experimental model that mimics the natural history of food tolerance and allergy induction.^29^ However, this response may vary depending on several factors, including the timing of tissue analysis. A compensatory rise in crypt cell proliferation, aimed at preserving the mucosal barrier, may produce a transient increase in villus height and crypt depth.^30^ In an animal model of diabetes,^30^ it was shown that the duodenum and ileum exhibited increased villus length and crypt depth compared to healthy animals, likely as a consequence of increased oxidative stress. It this study, in addition to the increased villus height in the duodenum and ileum of PG, PAaG, and AaG, the observed vascular congestion aligns with markers of tissue inflammation.^31^ Taken together, these results suggest that *Aa* JP2 may elicit atypical intestinal responses. This is supported by previous studies demonstrating that *Aa*, due to its multiple virulence factors, can invade tissues and evade the host immune response at sites of colonization.^4,8,9^

In the examined small intestine samples, a notable increase in goblet cell counts was observed, particularly in the jejunum and ileum. These cells are fundamental in maintaining mucosal epithelial homeostasis by secreting components of the mucosal barrier, which act as lubricants and help preserve a near-sterile epithelium. Additionally, they are crucial cellular components of the innate immune system, contributing to protection against external pathogens and debris.^32^ As key producers of protective mucus barriers, goblet cells dynamically respond to microbial challenges.^33^ This proliferation likely represents a compensatory immunoinflammatory response to *Aa* JP2-induced epithelial disruption. Interestingly, these findings contrast with those reported by da Costa, et al.^19^ (2024), who reported *Aa* JP2-mediated reductions in colonic leukocytes, highlighting potential segment-specific intestinal responses to oral pathogen colonization.

This study revealed significant associations between periodontal parameters, body mass, and gut mucosal morphology. Correlation analyses identified inverse associations between bone volume and jejunal goblet cell counts. Moreover, significant positive correlations were observed between bone loss (mean, mesial, buccal, or distal) and duodenal vascular congestion, as well as jejunal goblet cells. Vertical bone loss was also positively correlated with duodenal and ileal mass. Thus, increased periodontal destruction, as reflected by greater bone loss, was associated with intestinal morphological alterations. However, due to the bidirectional nature of correlation analyses, the directionality of these associations cannot be established. Cross-sectional studies have demonstrated that certain oral manifestations are a result of intestinal disease, while oral bacteria also appear to play an active role in the development of intestinal dysbiosis.^10,34^ Individuals with periodontitis, for example, present a distinct gut microbiota.^16^

An unexpected effect of *Aa* JP2 administration was observed in the dental pulp. Exposure to *Aa* JP2, with or without periodontitis, significantly increased vascular congestion compared to controls, which can be considered a precursor of pulp pathology.^35,36^ Notably, the current findings indicate that *Aa* JP2 alone induced pulpal abnormalities, demonstrating its independent pathogenic potential in this tissue. The effects of *Aa* JP2 on dental tissues (pulp and periodontal) in the absence of ligature-induced periodontitis (AaG) may be explained by its tropism for oral tissues,^11,12^ or by its ability to adhere to and interact with other periodontal pathogens.^13^ Once its presence is established, *Aa* JP2 is able to express its virulent factors, inducing tissue damage. Although this investigation did not test for the presence of *Aa* JP2 in oral tissues, its impact was evident in AaG. This group exhibited increased periodontal inflammatory infiltration and an increased number of congested blood vessels compared to CG. Notably, the inflammatory infiltrate profile observed in AaG more closely resembled that of PAaG than PG.

The use of ligature to induce periodontitis is a well-established method that typically elicits acute periodontal destruction, primarily measured by alveolar bone loss.^37^ In this study, an extended ligature period of 42 days deliberately employed to promote a chronic inflammatory response, which is expected to develop after approximately 15 days of induction.^37^ Previous investigations have shown that alveolar bone resorption remains relatively stable after the first 15 days of ligature placement, even when the experimental period is extended to 60 days.^37^ Moreover, substantial changes in the oral microbiota have been reported at 42 days of ligature-induced periodontitis.^38^ Therefore, the prolonged ligature period was chosen to increase the likelihood of detecting intestinal alterations resulting from sustained periodontal inflammation.

Our results suggest that periodontitis and/or administration of *Aa* JP2 may contribute to intestinal changes, highlighting the need for further investigation into these systemic interactions. Accordingly, caution is warranted when interpreting the observed correlations, given the animal model used and the sample size, particularly when extrapolating to clinical research.^39^ It should be noted that the sample size was ethically determined and calculated to be sufficient to address the primary research question. An additional limitation of this study is the exclusive use of male rats. Future investigations should include females to determine the generalizability of these findings, especially since previous research have reported a more pronounced periodontal or systemic^40^ inflammatory response in female rats following periodontitis induction. Another limitation is the lack of evaluation of *Aa* presence in oral tissues. Furthermore, analysis of fecal microbiota composition would be valuable to better characterize intestinal dysbiosis. Thus, future studies employing other experimental designs, including molecular approaches and the evaluation of other oral species, are required to further explore the potential bidirectional relationship involved in the oral–gut axis.

In conclusion, while *Aa* JP2 inoculation did not independently induce alveolar bone loss, it significantly increased periodontal inflammatory infiltrate regardless of periodontitis status. Both ligature-induced periodontitis and *Aa* JP2 exposure elicited distinct alterations in the small intestine, highlighting their independent systemic impacts. These findings underscore the need to investigate how oral pathogens may disrupt gut homeostasis beyond classical periodontal disease mechanisms.
